# Supplementary material for: In silico re-engineering of a neurotransmitter to activate KCNQ potassium channels in an isoform-specific manner
Source: Commun Biol. 2019 Nov 1;2:401. doi: 10.1038/s42003-019-0648-3 (PMC6825221; doi:10.1038/s42003-019-0648-3)
Supplement: Supplementary file 1 — Supplementary Information [file 42003_2019_648_MOESM1_ESM.pdf]

Supplementary Figures

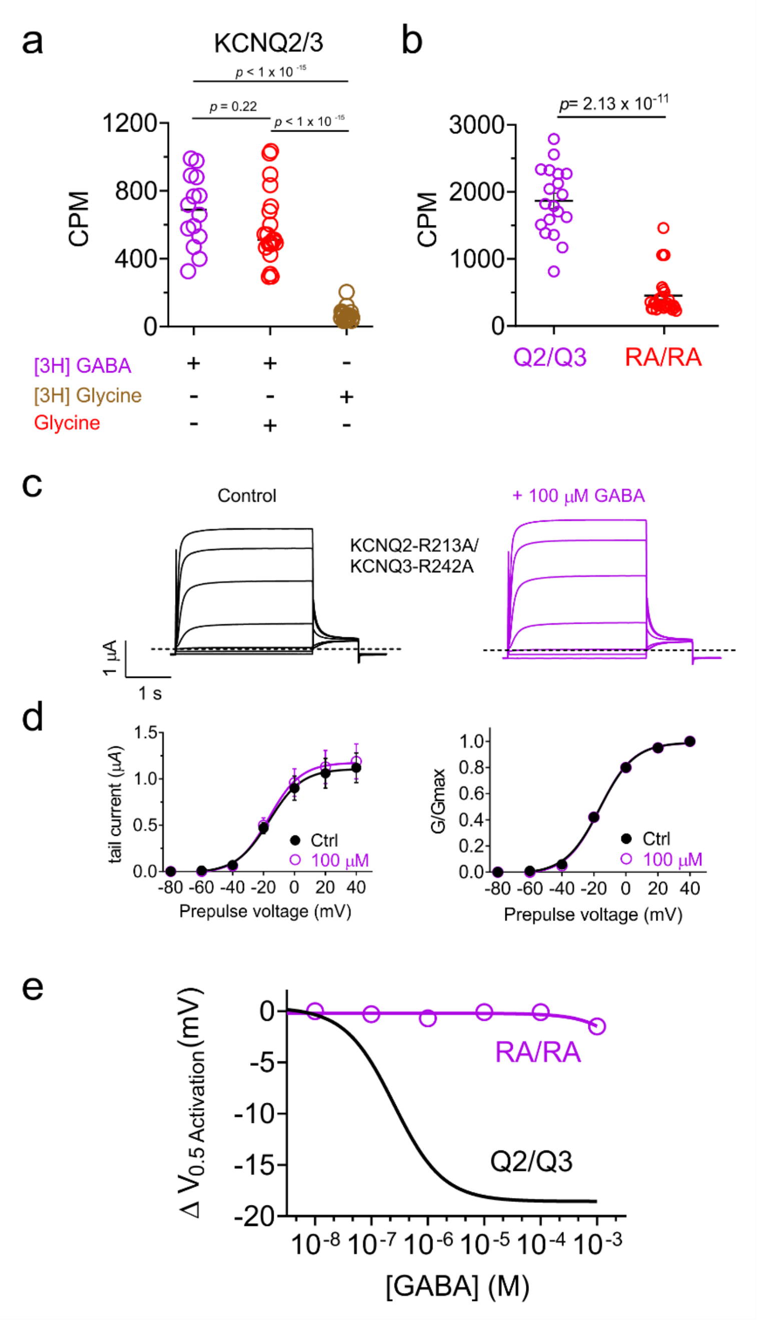

Supplementary Figure 1. GABA, 2FPG and 3FMSG utilize a similar KCNQ binding pocket that disallows glycine binding

All error bars indicate SEM.

a. [^{3}H]GABA or [^{3}H]glycine binding quantified in counts per minute (CPM, measured over 30 minutes) for oocytes expressing KCNQ2/3 in the absence or presence of cold glycine as indicated; *n* = 15-20. Each point = 1 oocyte.

b. [^{3}H]GABA quantified in counts per minute (CPM, measured over 30 minutes) for oocytes expressing wild-type KCNQ2/3 (Q2/Q3) or KCNQ2-R213A/KCNQ3-R242 (RA/RA) as indicated; *n* = 19-29. Each point = 1 oocyte.

c. Functional effects of KCNQ2-R213A/KCNQ3-R242 mutations on KCNQ2/3 heteromeric channel GABA sensitivity. Mean traces shown in the absence (Control) or presence of GABA (100 μM); *n* = 5.

d. Mean raw tail current and normalized tail current (G/Gmax) for traces as in panel c; *n* = 5.

e. GABA concentration versus the induced shift in $V_{0.5\ Activation}$ for wild-type KCNQ2/3 (Q2/Q3) (data from Figure 1e) versus KCNQ2-R213A/KCNQ3-R242 (RA/RA); *n* = 5.

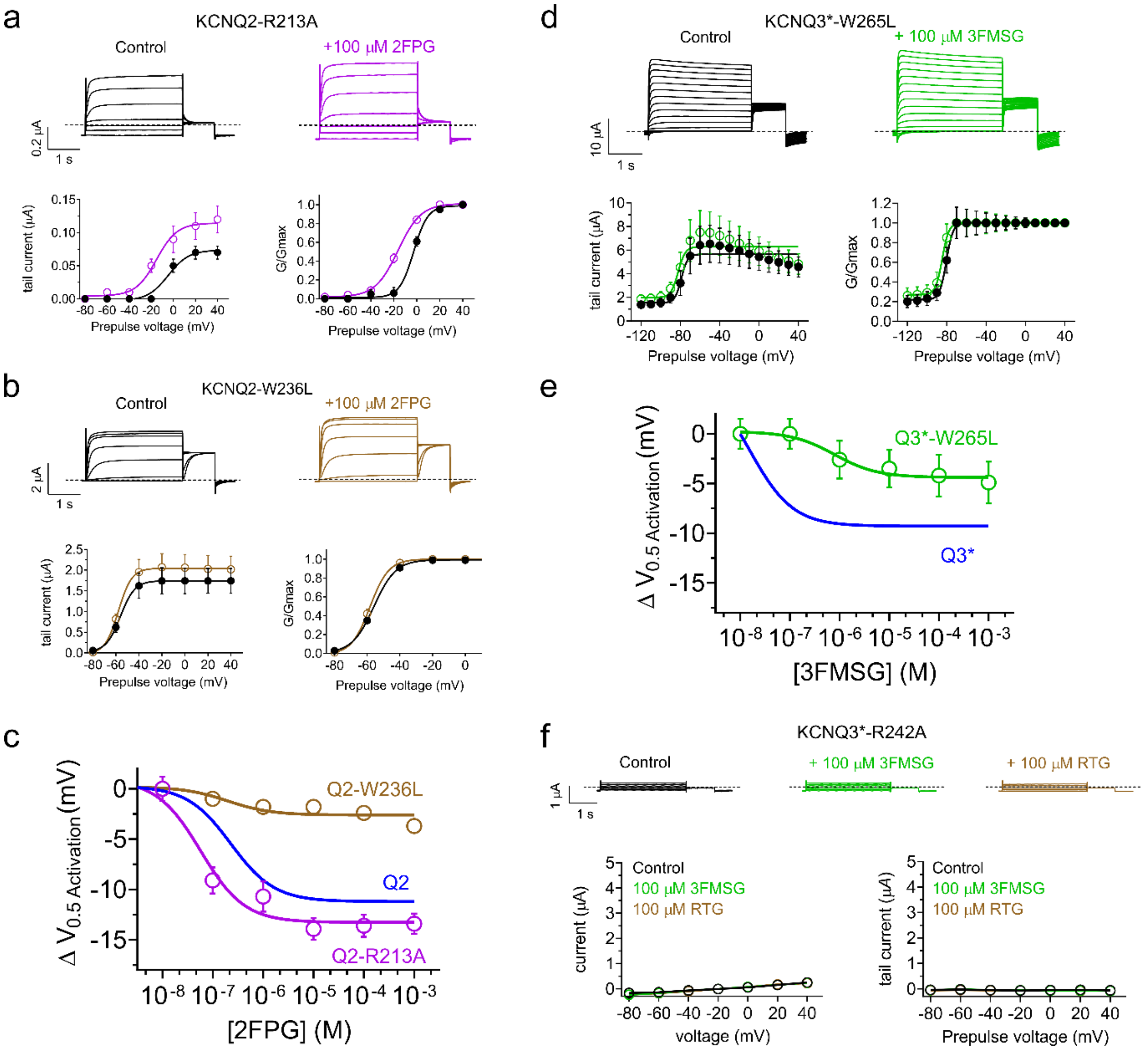

Supplementary Figure 2. Effects of mutations on homomeric channel sensitivity to 2FPG and 3FMSG

All error bars indicate SEM.

a. Mean traces, raw tail current and G/Gmax showing effects of 2FPG (100 μM) on KCNQ2-R213A (*n* = 4).

b. Mean traces, raw tail current and G/Gmax showing effects of 2FPG (100 μM) on KCNQ2-W236L (*n* = 5).

c. 2FPG concentration versus the induced shift in $V_{0.5\ Activation}$ for wild-type KCNQ2 (Q2) (data from Figure 4c) versus Q2-R213A and Q2-W236L; *n* = 4-5.

d. *M*ean traces, mean raw tail current and G/Gmax showing effects of 3FMSG (100 μM) on KCNQ3*-W265L (*n* = 5).

e. 3FMSG concentration versus the induced shift in $V_{0.5\ Activation}$ for wild-type KCNQ3* (Q3*) (data from Figure 4i) versus Q3*-W265L; *n* = 5.

f. Mean traces, mean raw prepulse current and raw tail current showing lack of effects of 3FMSG or retigabine (100 μM) on the nonfunctional KCNQ3*-R242A channel (*n* = 5).
