# Supplementary material for: In silico re-engineering of a neurotransmitter to activate KCNQ potassium channels in an isoform-specific manner
Source: Commun Biol. 2019 Nov 1;2:401. doi: 10.1038/s42003-019-0648-3 (PMC6825221; doi:10.1038/s42003-019-0648-3)
Supplement: Supplementary file 2 — Description of Additional Supplementary Files [file 42003_2019_648_MOESM2_ESM.docx]

Description of Additional supplementary Items

One Excel file (Supplementary Data 1) containing 68 data tables referred to in the text.
